# Multiple synchronous primary malignancies induced by benzene exposure: a case report

**DOI:** 10.1186/1745-6673-4-7

**Published:** 2009-04-16

**Authors:** Pingli Wang, Gensheng Zhang, Huahao Shen

**Affiliations:** 1 Department of Respiratory Disease, Second Affiliated Hospital of Medical School of Zhejiang University. Hangzhou, 310009, PR China

## Abstract

**Background:**

Chronic exposure to high concentrations of benzene is usually associated with the development of haematological diseases. However, solid tumors induced by benzene exposure are less frequent.

**Case presentation:**

We present an unusual case of triple synchronous primary malignancies most likely induced by occupational benzene exposure in a male patient. This spray painter was diagnosed as chronic aplastic anemia in his 21 years old after exposing to high concentration of benzene for three years. Then he was treated with glucocorticoid for four years. 40 years later, this patient developed three synchronous primary neoplasms with three different histologies including a basaloid squamous cell carcinoma of the esophagus, primary hepatocellular carcinoma, and well-differentiated squamous cell carcinoma of the gum.

**Conclusion:**

This case reminds us that the occurrence of solid tumors should be monitored in workers with occupational history linked with a high concentration exposure to benzene, though it's rarely happened.

## Background

Chronic exposure to high concentrations of benzene in humans is usually associated with the development and progression of leukaemia and other haematological diseases [1-3]. Less frequently, solid tumors induced by benzene exposure may occur.

We present a rare case of triple synchronous primary malignancies with chronic aplastic anemia induced by strongly related occupational benzene exposure in a male patient. This patient had three primary neoplasms with three different histologies including a basaloid squamous cell carcinoma of the esophagus, primary hepatocellular carcinoma, and well-differentiated squamous cell carcinoma of the gum. These neoplasms simultaneously occurred 40 years after benzene exposure.

## Case presentation

A 61-year old male patient was admitted to our hospital in May 2007 with a solid mass in the right-inferior gum for almost a year and progressive enlargement in latest two weeks. He had no respiratory or gastrointestinal complaints, and he denied any weight loss. In this spray painter, chronic aplastic anemia was diagnosed when he was 21 years old, after a previous three-year exposure to high concentration of benzene. Consequently, he was treated with glucocorticoid for four years. After that this patient lived together with his brother in Hangzhou, and he only did some houseworks. He had never smoked nor been exposed to smoking environment. 40 years later, three synchronous primary neoplasms including a basaloid squamous cell carcinoma of the esophagus, primary hepatocellular carcinoma and well-differentiated squamous cell carcinoma of the gum were diagnosed in this patient.

**Figure 1:**
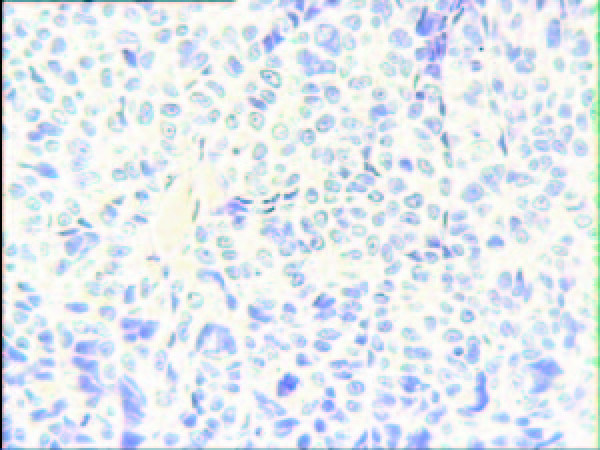
**Microscopic findings of biopsy specimen obtained by esophagogastroscopy showing atypical cells diagnosed as basaloid squamous cell carcinoma (H&E stain ×400)**.

In late October 2006, he developed progressive dysphagia and a barium swallow and computed tomography (CT) of esophagus showed an esophageal neoplasm. Esophagogastroscopy and histopathological analysis of the biopsy specimen revealed a basaloid squamous cell carcinoma (Figure 1). The patient underwent a radical operation of esophageal carcinoma. CT of abdomen showed a cycloid hypodense lesion with a clear margin in the left liver. He was suggested follower-up for the lesion in the liver. The repeated CT of abdomen two month later showed a little enlargement of the lesion. The patient had no family history of malignancy. He had also no history of hepatitis.

**Figure 2:**
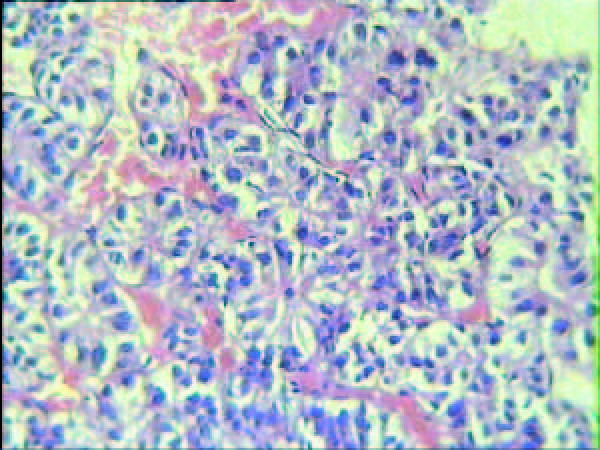
**Microscopic findings of a biopsied hepatic specimen showed primary hepatocellular carcinoma (H&E stain ×400)**.

**Figure 3:**
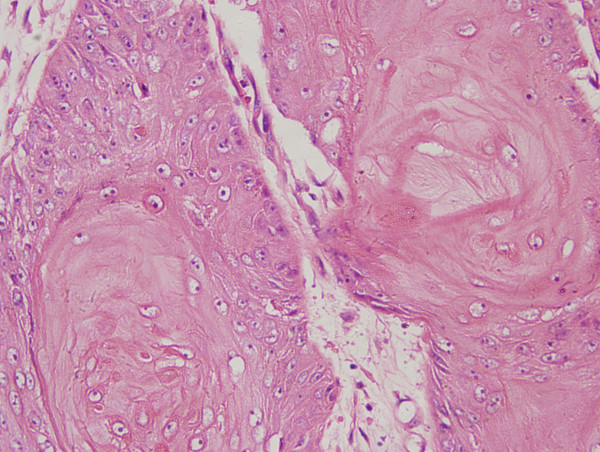
**Excised tissue of the gum showed the histologic appearance of a well-differentiated squamous cell carcinoma**. (H&E stain ×400).

On physical examination, a painless rigid mass (2 × 1.5 cm) was found on right-inferior gum near the first molar. Chest X-ray and electrocardiogram were unremarkable. The bone marrow puncture and biopsy was performed and showed the marrow failure. Hepatic ultrasonography revealed two low echoic masses at level S2-3 (maximum diameter 3.9 cm), and the entire liver parenchyma had heterogeneous echogenicity. Histopathological analysis of the biopsy specimen revealed primary hepatocellular carcinoma (Figure 2). A gum biopsy from the mass was performed, and showed well-differentiated squamous cell carcinoma (Figure 3). So the patient underwent a radical operation for the mass on June 23, 2007. Neither adjuvant chemical therapy nor radiation therapy was given during the period of hospitalization. The patient was discharged on July 16, 2007. Five months later, he died of massive hemorrhage of gastrointestinal tract induced by thrombocytopenia.

## Discussion

This is a case report of a patient diagnosed with three synchronous primary tumors and who had previous occupational benzene exposure for 3 years (1964–1966) and an acute benzene poisoning.

Multiple primary malignant neoplasms (MPMN) in a single patient are not frequent. The majority of MPMN occurring in multiple organs are metachronous, while the synchronous tumors, which are defined that two or more primary tumors are diagnosed within 6 months of the first primary tumor, are less frequent. Our case had three such synchronous primary neoplasms. Regarding the aetiology of multiple primary malignancies, several factors have been incriminated: genetic, hormonal (e.g. sex steroids), environmental, iatrogenic (e.g. chemotherapy, radiation therapy, hormonal and immunosuppressive medications) and immunologic factors (the loss of immunity). In this case, the benzene exposure history was considered as the most possible causative factor for his MPMN.

Benzene and its metabolites are highly clastogenic[4]. Chronic exposure to high concentrations of benzene in humans is associated with hematotoxicities, including pancytopenia, aplastic anemia, myelodysplasia, and acute myeloid leukemia [5-7]. The hematotoxicity of benzene is related to the amount and duration of exposure. At high levels of exposure (air concentration > 100 ppm), the incidence of aplastic anemia is approximately 1/100 individuals exposed; at lower levels of exposure (10–20 ppm), this drops abruptly to approximately 1/10,000[8]. Although the exact benzene exposure level for this patient could not be determined, some earlier studies of benzene exposure provided useful information [5,6]. In a NCI-CAPM study, Dosemeci et al provided that the expose level for "spray painter" at a Shanghai bicycle factory during 1965–1969 was 331 mg/m^3 ^or 104.1 ppm[5]. The author also implicated that some engineering changes took place over the years, resulting in lower exposure levels. Thus, exposure for spray painters before 1965 should be the same as, or more likely, higher than those recorded during 1965–1969. Wong also pointed out the actual level might be higher than that in the NCI-CAPM study[6]. According to these studies, we can estimate that the benzene exposure level for this patient would be much higher at that time. It seemed almost certain that his benzene exposure history played a key role in the occurring of aplastic anemia.

The relationship between benzene exposure and solid tumor is not known well. According to a previous study in 12 cities in China[9], a small increase was observed in total cancer mortality among benzene-exposed compared with unexposed workers (relative risk [RR] = 1.2). Statistically significant excess was noted for lung cancer (RR = 1.4), but less than leukemia (RR = 2.3). Some studies showed that there was no indication of increased incidences of solid tumors for chronic benzene exposed workers or children [10-12]. However, animal experimental results showed that several solid tumors occured in the Zymbal gland, oral and nasal cavities, liver, and mammary gland of Sprague-Dawley rats following chronic, high-dose administration of benzene, which was thought to be caused by activation of toxic metabolites that can interact with DNA, and form covalent adducts[13].

Treatment for aplastic anemia is another possible cause of multiple tumors. Long-term administration of androgenic steroids for aplastic anemia was reported in some cases correlating to multiple neoplasms[14,15]. Our patient had never taken androgen but glucocorticoid to treat the aplastic anemia. To our knowledge, there was no case reported that glucocorticoid treatment for aplastic anemia resulted in the occurrence of MPMN.

## Conclusion

This is a rare case manifesting a combination of three synchronous primary malignant neoplasms and chronic aplastic anemia most likely induced by occupational benzene exposure. It reminds us that the occurrence of solid tumors should be monitored in workers with occupational history linked with a high concentration exposure to benzene, though it's rarely happened.

## Competing interests

The authors declare that they have no competing interests.

## Authors' contributions

PW participated in the study design and coordination as well as drafting of the manuscript, GZ helped to data collection and draft the manuscript, HS conceived of the study, participated in coordination and drafted the manuscript. All authors read and approved the final manuscript.

## Consent

Written informed consent was obtained from the patient's brother (legal guardian) for publication of this case report and any accompanying images. A copy of the written consent is available for review by the Editor-in-Chief of this journal.
